# Scabies in General Practice

**Published:** 1908-04-11

**Authors:** 


### DERMATOLOGY.

### SCABIES IN GENERAL PRACTICE.

Scabies is met with daily in hospital practice. The general practitioner does not see it so often, and when he does it usually comes upon him unawares, so that he not seldom mistakes it for eczema. He does not expect to find it in his better-class patients. But it may occur, and he should be prepared for it. In fact, in any case of generalised pruritus, the question of scabies should cross his mind. An infant may contract it from a nursemaid; it may break out among the children in a convalescent home, or other institution; it may occasionally be conveyed from one person to another by the common use of tools, writing implements, etc.; or it may be contracted as a venereal disease. The affection may be from the first wrongly diagnosed as eczema; or sometimes, scabies is suspected, and sulphur applications are ordered, but not efficiently applied, and then the first view is abandoned, and the case is regarded as a very obstinate form of eczema. The only way to avoid these errors is always in the presence of a case of general itching, or of a pruritus about the hands and forearms, to think of scabies, and to examine for the initial and characteristic lesion of scabies, the burrow of the acarus; and if any lesion suggesting a burrow is present to search for the acarus itself. If, in a doubtful case, this is once found, the diagnosis is certain.

In order to find the acarus a watchmaker's magnifying glass is to be employed, as in the diagnosis of ringworm. First of all one examines carefully around and between the fingers, the sides of the palms, and the wrists. The burrow is seen as a wavy line, black as a rule, but sometimes, in cleanly persons, white. It represents the path of the female acarus in the upper layers of the epidermis, where her eggs are deposited. Sometimes the burrow is seen passing over, near one end, a tiny vesicle; but this vesicle is disregarded in the search for the acarus. With a little care the latter can easily be seen at one end of the burrow as a minute raised smooth whitish rounded body, under the glass looking like a tiny pebble imbedded in the skin, and just visible to the naked eye. With the point of a needle this little speck is easily dug out. It clings to the needle-point and can be removed to a slide and examined in potash solution under the microscope. A picture of the acarus is to be found in most works on dermatology, and it need not be described here. Sometimes, either because the patient is accustomed to wash his hands frequently, or because his work necessitates their being constantly in water (or, in hospital patients, because the burrows are often marked by secondary impetiginous pustules and crusts), there may be great difficulty in finding the acarus. In children it can often be found more readily on the sides of the feet. In male adults burrows are commonly on the penis, where they are almost invariably inflamed, and appear as elongated red swellings.

It must be remembered that scabies in infants often gives rise to a generalised eczematous eruption, and that the face does not escape, as in adults. In fact, an eczematous and pruritic eruption in an infant should always make one suspect scabies, and if the mother or the nurse is also suffering from pruritus the diagnosis is almost certain. Such cases, however, are seldom met with except among the poor. In general practice a universal pruritus in a child is most commonly the result of lichen urticatus, but the possibility of scabies must be borne in mind. It may be very difficult to distinguish between these two affections, and often this can only be done by discovering the primary lesion: in scabies, the burrow; in lichen urticatus, the papule-centred wheal.

*Treatment.*—The old-fashioned remedy of sulphur in the form of an ointment still remains the most generally useful application in scabies. But in order to effect a cure rapidly, and without the production of a subsequent sulphur dermatitis it is necessary to carry out the treatment thoroughly at the first, and not to continue it too long. The patient should take a warm bath, and he should soak in this for a quarter of an hour, and while in the bath he should soap himself or be soaped, all over. After the bath the ointment should be well rubbed over the whole body (excluding the head and face), and the patient should sleep in the undergarments which he has worn during the day. In the morning he may take a bath to remove the ointment, and for the two following nights he must carry out the same treatment. The pruritus will often continue after the acari have thus been destroyed, but the sulphur applications must not be used again. A lotion of liq. carbonis detergens (℥j to a pint of water, or, better still, in calamine lotion) will relieve the itching which is felt after the course of sulphur treatment. In some cases a second shorter course may be necessary.

The following is a suitable prescription for the sulphur ointment in the case of an adult:—

| | |
|---|---|
| Sulphur ... ... ... ... ... | ʒj. |
| Adipis benzoat ... ... ... ... | ʒj. |
| Ol lavandulæ ... ... ... ... ... | ♏xv. |

